# The Ins and Outs of Herpesviral Capsids: Divergent Structures and Assembly Mechanisms across the Three Subfamilies

**DOI:** 10.3390/v13101913

**Published:** 2021-09-23

**Authors:** Elizabeth B. Draganova, Jonathan Valentin, Ekaterina E. Heldwein

**Affiliations:** 1 Department of Molecular Biology and Microbiology, Tufts University School of Medicine, Boston, MA 02111, USA; elizabeth.draganova@tufts.edu; 2 Department of Chemical Engineering, University of Florida, Gainesville, FL 32603, USA; valentinj@ufl.edu

**Keywords:** herpesvirus, capsid, assembly, capsid associated-tegument complex (CATC)

## Abstract

Human herpesviruses, classified into three subfamilies, are double-stranded DNA viruses that establish lifelong latent infections within most of the world’s population and can cause severe disease, especially in immunocompromised people. There is no cure, and current preventative and therapeutic options are limited. Therefore, understanding the biology of these viruses is essential for finding new ways to stop them. Capsids play a central role in herpesvirus biology. They are sophisticated vehicles that shelter the pressurized double-stranded-DNA genomes while ensuring their delivery to defined cellular destinations on the way in and out of the host cell. Moreover, the importance of capsids for multiple key steps in the replication cycle makes their assembly an attractive therapeutic target. Recent cryo-electron microscopy reconstructions of capsids from all three subfamilies of human herpesviruses revealed not only conserved features but also remarkable structural differences. Furthermore, capsid assembly studies have suggested subfamily-specific roles of viral capsid protein homologs. In this review, we compare capsid structures, assembly mechanisms, and capsid protein functions across human herpesvirus subfamilies, highlighting the differences.

## 1. Introduction

Herpesviruses are enveloped DNA viruses that infect almost all vertebrates and some invertebrates [1]. Nine herpesviruses, classified into three subfamilies (denoted in italics), infect humans. They are herpes simplex virus types 1 and 2 (HSV-1 and HSV-2) and varicella-zoster virus (VZV) (*Alphaherpesviruses*); human cytomegalovirus (HCMV) and human herpesvirus types 6A, B and 7 (HHV-6A/B and HHV-7) (*Betaherpesviruses*); and Epstein–Barr virus (EBV) and Kaposi’s sarcoma herpes virus (KSHV) (*Gammaherpesviruses*) [2]. These viruses establish lifelong latent infections within the host and can cause a range of diseases that can be particularly severe in the immunocompromised people. Current antivirals do not eliminate these infections, and vaccines are available against only VZV. Therefore, a detailed understanding of the biology of these viruses is essential for developing new antiviral strategies.

**Figure 1:**
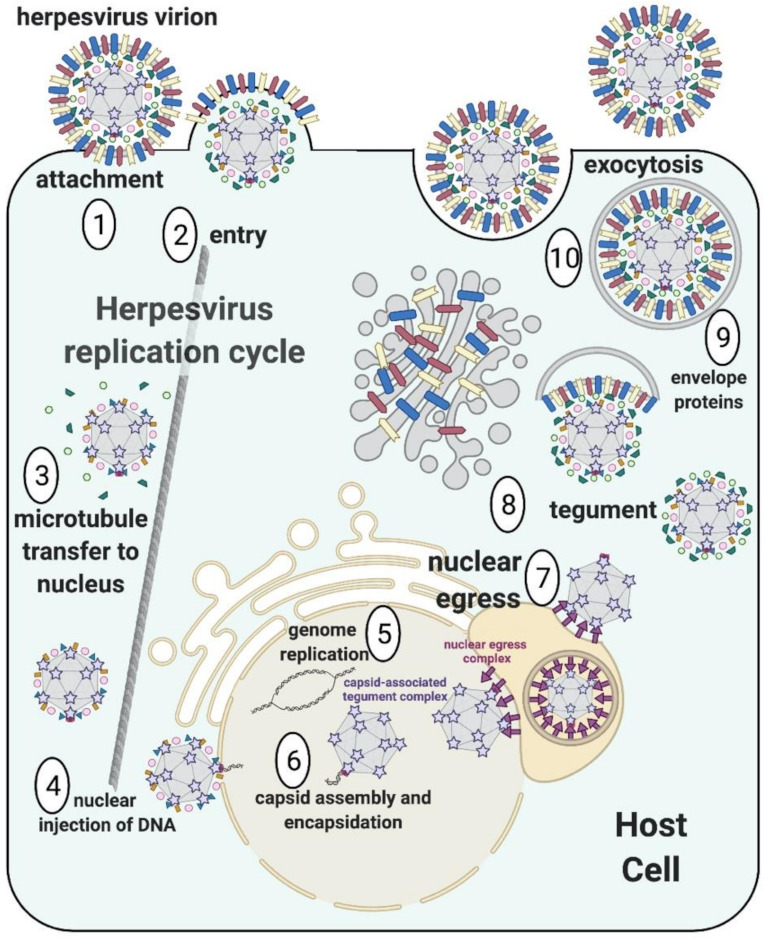
Overview of the herpesvirus replication cycle. An incoming virion attaches to the host cellular membrane (1) and fuses with it, releasing the capsid into the cytoplasm (2). Some of the outer protein layer, termed the tegument, dissociates upon entry, and the capsid traffics to the nucleus via microtubules (3). The capsid then docks at the nuclear pore and releases the DNA genome into the nucleus (4), where the incoming genome undergoes replication (5). Progeny genomes are packaged into capsids decorated with auxiliary proteins (e.g., the capsid-associated tegument complex) (6) and bud through the nuclear envelope with the help of the nuclear egress complex into the cytoplasm (7). Capsids gain a tegument layer (8) and acquire a protein-studded lipid envelope by budding at vesicles derived from *trans*-Golgi network and endosomes (9). The mature virions use the exocytic pathway to exit the cell (10). The figure was created with BioRender.com, accessed on 19 September 2021.

Capsids play a central role in the biology of herpesviruses. In addition to containing and protecting the viral genome, the capsid serves as a vehicle. Upon entry, the capsid interacts with the cellular motor proteins to transport the incoming genomes from the cellular periphery to the nucleus (Figure 1). Once progeny genomes are replicated and encapsidated in the nucleus (reviewed in [3,4]), capsids traffic them across the nuclear envelope to the cytoplasmic viral assembly sites with the help of the nuclear egress complex [5]. To fulfill these functions, herpesviral capsids employ multifunctional proteins and have evolved specific properties.

Herpesviral capsids are ~125 nm in diameter and have icosahedral symmetry (T = 16), which is characterized by 20 faces and 12 vertices (Figure 1) (reviewed in [6,7]). The capsid shell is composed of two types of building blocks, termed capsomers, that are formed by the major capsid protein (MCP). The MCP arranges into either hexons, which form the faces of the icosahedron, or pentons, which are located at 11 out of the 12 vertices. The 12th vertex houses the viral genome portal, formed by twelve copies of the portal protein. Additional structural components are the small capsid protein (SCP) and the triplexes. During assembly, capsids also utilize a protein scaffold and protease, two proteins with overlapping gene sequences, which bind the MCP. However, only the protease is found in the final mature capsid. The main capsid building blocks (MCP, SCP, triplexes, protease, and scaffold) are conserved across human herpesviruses. Finally, additional, so-called auxiliary, proteins, recruited to the capsid during assembly in the nucleus (Figure 1), decorate the exterior of the capsid shell.

Recent cryo-electron microscopy (cryo-EM) reconstructions of herpesviral capsids from the three subfamilies not only revealed the overall similarities in the capsid structures but also the subfamily-specific differences in the auxiliary proteins [8,9,10,11,12,13,14,15,16]. In this review, we compare capsid structures and assembly mechanisms across the three subfamilies of human herpesviruses. We also discuss the roles of the auxiliary capsid proteins in viral replication, highlighting both conserved and subfamily-specific roles.

## 2. Capsid Assembly

### 2.1. Overview of Capsid Components and Experimental Assembly Systems

**Figure 2:**
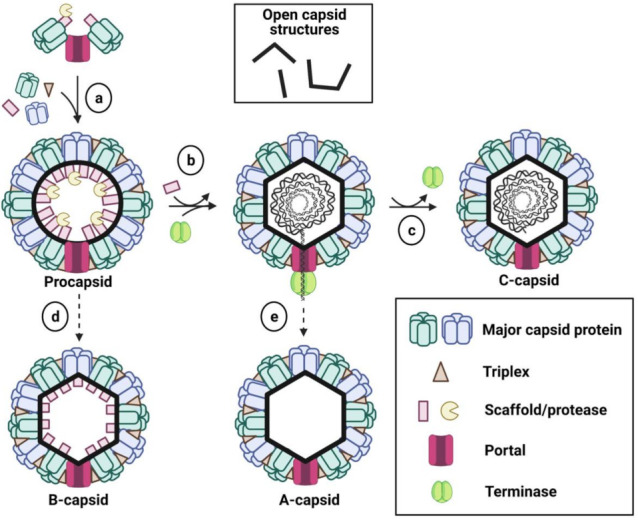
Herpesvirus capsid assembly pathway. (a) Capsid assembly is thought to be nucleated by the portal complex that interacts with the MCP, triplexes, or scaffold. The MCP and triplexes assemble around the scaffold into a spherical procapsid. (b) The terminase complex binds to the portal and the protease cleaves the scaffold. The capsid is angularized, the scaffold is extruded, and the DNA genome is packaged into the capsid. (c) The DNA is then cleaved and the terminase is released, resulting in the formation of a mature C-capsid. B-capsids (d) and A-capsids (e) are thought to be the by-products of assembly. B-capsids form whenever the scaffold fails to be released from the capsid whereas A-capsids form if the scaffold is released but the DNA does not get packaged. The top inset illustrates the open capsid structures observed in some of the capsid assembly experiments described in the text. For clarity, the SCP and auxiliary proteins are not shown. The figure was created with BioRender.com, accessed on 10 July–19 September 2021.

Capsids assemble in the nucleus from the MCP, triplexes, SCP, protease, scaffold, and portal proteins (Figure 2) (reviewed in [6,17]). (Additional, so-called auxiliary, proteins are also found on the capsids, but they are less conserved across subfamilies and are discussed in more detail in Section 3). Capsid proteins and the genes encoding them have different names across herpesviruses, which can complicate comparisons. For clarity, we will be referring to each of the capsid protein homologs across subfamilies using names that describe their major functions. Subfamily-specific gene and protein names are provided in Table 1 for reference. Current herpesviral capsid assembly models originate from extensive studies of HSV-1 capsid assembly followed up by studies of EBV and KSHV assembly. Capsid assembly has been largely studied using intact insect cells that can produce capsids upon infection with recombinant baculoviruses engineered to express capsid proteins of HSV-1 [18,19,20], EBV [21], or KSHV [22]. In the case of HSV-1, capsid assembly has also been studied using lysates of infected insect cells described above [23,24] or purified capsid proteins [25]. The resulting observations suggest that both alpha- and gammaherpesvirus subfamilies generally follow a similar assembly pathway, with a few notable differences (described below and summarized in Table 2). Betaherpesvirus capsid assembly has not yet been investigated in detail but likely also uses a conserved pathway.

### 2.2. Capsid Assembly Pathway

Capsid assembly is thought to be nucleated by the portal complex and mediated by the MCP and triplexes that form a spherical procapsid around the scaffold and the protease (Figure 2) [25,26,27]. Concomitantly, concatemeric DNA is cleaved into unit-length genomes and brought to the capsid for packaging by the terminase complex, a group of three viral proteins (UL15, UL28, and UL33 in HSV-1 [28]) essential for DNA packaging [6,17]. Binding of the terminase complex to the portal triggers the protease to cleave the scaffold, which causes angularization of the procapsid [24,29,30,31,32]. A region of the MCP on the capsid exterior has also been implicated in triggering the angularization of the procapsid [33]. Upon angularization, the SCP binds the capsid [30].

The scaffold is then released, followed by ATP-driven packaging and cleavage of the DNA genome by the terminase complex (reviewed in [6,34]). Here, UL15 provides the ATPase and nuclease activity whereas UL28 is responsible for site-specific binding of the packaging signal sequence within the viral DNA genome. UL33 stabilizes the complex. Upon completion of DNA packaging, the terminase complex is thought to dissociate [35,36,37], and the mature, DNA-containing C-capsid is formed. Two additional types of angularized capsids lacking the DNA are observed in infected cells, the A- and B-capsids. The A-capsids are empty, whereas the B-capsids retain the cleaved scaffold. Both the A- and B-capsids are thought to represent dead-end by-products of assembly (Figure 2) (reviewed in [38]). Although all three types of angularized capsids, along with the procapsids, are found in the infected nucleus, the C-capsids are predominantly found in the cytoplasm [39]. This implies the existence of a selection mechanism that favors the translocation of the mature DNA-filled capsids from the nucleus into the cytoplasm, which ensures the efficient production of infectious virions.

### 2.3. Sub-Family-Specific Roles of Capsid Proteins in Alphaherpesvirus Assembly

#### 2.3.1. MCP, Triplexes, the Formation of Procapsids, and the Portal

In the case of HSV-1, B-capsids can assemble when MCP, SCP, triplexes 1 and 2, scaffold, and protease are overexpressed in insect cells infected with recombinant baculoviruses or in vitro when cell lysates from these experiments are mixed at 28 °C for 12 h [19,20,23]. These capsids are very similar in appearance to the B-capsids obtained from HSV-1-infected cells except that they have less SCP. “Open” crescent-shaped capsid structures (Figure 2, inset)—presumably, capsid assembly intermediates that failed to form a closed shell—were also observed in both experimental systems. These were more common in the in-vitro assembly system, suggesting that capsid formation in vitro is less robust than in infected cells. Alternatively, capsids formed in vitro are less stable and, therefore, less likely to withstand the sedimentation process used to isolate them. Overall, the three studies found that the MCP and triplexes were required for B-capsid assembly [19,20,23] (Table 2), similarly to what was observed during infection with HSV-1 mutant viruses lacking either the MCP, triplex 1, or triplex 2 proteins [40,41]. Therefore, the MCP and the triplex proteins are the essential components of capsid assembly regardless of the experimental system.

**Table 2:** Comparison of the effects on capsid assembly in the absence of specific capsid shell proteins (e.g., ΔMCP) across human herpesvirus subfamilies under different experimental conditions (in vitro, in insect cells infected with recombinant baculoviruses, or in herpesvirus-infected cells). Boxes with dashed lines indicate a lack of published data.

	Alpha			Beta	Gamma	
	HSV-1			HCMV	EBV/KSHV	
∆Protein	In Vitro	Insect Cells	Infected Cells	Infected Cells	Insect Cells	Infected Cells
**∆MCP**	No capsid formation [23]	No capsid formation [19,20]	No capsid formation [40]	No viral replication (capsid assembly not investigated) [42]	No capsid formation [21,22]	--
**∆SCP**	B-capsids [23]	B-capsids [19,20]	A-, B- and C-capsids form in Vero cells and ocular mouse model ^b^ [43]	B-capsids [44]	No capsid formation [21,22]	Severe defect in capsid formation; some empty capsids (KSHV) [45,46]
**∆Triplex 1**	No capsid formation [23]	No capsid formation [19,20]	No capsid formation [41]	No viral replication (capsid assembly not investigated) [42]	No capsid formation [21,22]	--
**∆Triplex 2**	No capsid formation [23]	No capsid formation [19,20]	No capsid formation [40]	No viral replication (capsid assembly not investigated) [42]	No capsid formation [21,22]	--
**∆Scaffold**	--	Empty angularized capsids and open shells [19,20]	B-capsids [47]	Some closed capsids of unknown identity; open capsid shells [48]	No capsid formation; only open capsid structures [21,22]	--
**∆Protease**	Capsids form at a reduced yield; capsid type was not determined ^a^ [23]	Angularized capsids, similar in appearance to B-capsids [19,20]	Only procapsids; these are capable of maturation if isolated and incubated at room temperature [30,49]	Angularized capsids, similar in appearance to B-capsids with dense cores [50]	B-capsids [21,22]	Only closed spherical procapsids (KSHV) [51]
**∆Scaffold + ∆Protease**	No capsid formation [23]	No capsid formation; only open capsid structures [19,20]	No capsid formation; only open capsid structures [52]	--	No capsid formation; only open capsid structures [21,22]	--
**∆Portal**	B-capsids [23]	B-capsids [19,20]	B-capsids either when deleted [53] or portal/scaffold interactions perturbed [54,55]	--	B-capsids [21,22]	--

^a^ Procapsids form predominantlyfrom a mixture of purified MCP, triplexes, and scaffold [25]. ^b^ VZV capsid assembly is perturbed in a melanoma cell line (only empty spherical capsids form) [56].

Angularized HSV-1 capsids form from a spherical procapsid precursor in infected cells (Figure 2) [24,30,57]. The existence of a procapsid precursor was confirmed in vitro by mixing insect cell lysates containing MCP, triplexes, scaffold, and protease, produced from recombinant baculoviruses, and monitoring assembly over time [24]. Spherical procapsids formed within 90 min at 28 °C and matured into B-capsids after 8 h. Additionally, procapsids extracted from the lysates could mature into B-capsids upon incubation at 37 °C. Collectively, these in vitro findings suggest that B-capsids form from a procapsid precursor and that the angularization process does not require any host proteins or viral proteins other than MCP, triplexes, scaffold, and protease [23,24].

While the portal is dispensable for B-capsid formation in the recombinant baculovirus/insect cell system or in vitro [19,20,23], no capsids lacking the portal have yet been isolated from HSV-1-infected cells. In HSV-1-infected cells, capsid assembly is thought to be nucleated by interactions of the portal complex with the MCP, triplexes, or scaffold [25,26,27]. In vitro capsid assembly experiments in the presence of the portal produce portal-containing capsids that resemble B-capsids isolated from HSV-1-infected cells [58]. Moreover, incorporation of the portal into the B-capsids in vitro required interactions between the portal and the scaffold [59]. Similar findings in HSV-1-infected cells [54,55] suggest that the predominant capsid assembly mechanism requires interactions of the portal with the scaffold. In the absence of the portal, capsid assembly in the recombinant baculovirus/insect cell system or in vitro must be nucleated through an alternative mechanism.

#### 2.3.2. Protease and Scaffold

In in-vitro HSV-1 assembly experiments, capsids fail to form if both the scaffold and the protease are missing (Table 2) [23]. Likewise, in insect cells infected with recombinant baculoviruses expressing only the MCP, triplex 1, and triplex 2 proteins of HSV-1, only “open” crescent structures were observed (Figure 2 inset) [19,20]. Similar crescent structures were also observed in cells infected with an HSV-1 mutant virus lacking both the scaffold and the protease genes [52]. Furthermore, it has been shown that the C-terminal ends of the scaffold and protease proteins bind the MCP [18]. Perturbation of these interactions impairs the formation of closed capsid shells in infected cells [60]. Therefore, interactions between the scaffold/protease and the MCP are required for the closure of the procapsid shell.

In the absence of the HSV-1 protease, capsids similar to B-capsids but with denser cores were formed in the recombinant baculovirus/insect cell system [19,20] and in vitro [23] (Table 2). These capsids had dense cores, presumably, because the scaffold was not cleaved. Additionally, similar capsids were observed in cells infected with a temperature-sensitive (ts) protease mutant HSV-1 at a nonpermissive temperature [61]. Switching to a permissive temperature restored protease activity, allowing scaffold cleavage and DNA packaging to resume. In cells infected with protease-null mutant HSV-1, only procapsids were observed [30,49]. The capsids formed in cells infected with the ts protease mutant HSV-1 at the nonpermissive temperature also corresponded to procapsids [30]. These findings raise the possibility that the capsids formed in the absence of the protease in both insect cells [19,20] and insect cell lysates [23] could represent a mixture of procapsids and B-capsids.

In vitro, combining purified MCP, triplexes, and scaffold at 37 °C for 4 h produced mostly procapsids with very few angularized capsids [25]. However, longer incubation times might have increased the yield of the angularized capsids. Indeed, procapsids isolated from cells infected with protease-null mutant HSV-1 slowly become angularized in vitro at 21 °C after 60–72 h incubations [30]. In contrast, in the presence of the protease, procapsids isolated from insect cell lysates matured more rapidly in vitro, within 8 h [24]. These observations suggest that while the protease may not be required for angularization, it may accelerate it.

In the absence of the HSV-1 scaffold, in the recombinant baculovirus/insect cell system, both empty angularized capsids and open crescent-shaped shells were seen (Table 2) [19,20]. This suggests that the protease can function as a scaffold, even if inefficiently. The role of the scaffold in the in vitro cell lysate experiments has not yet been tested. In cells infected with the scaffold-null mutant HSV-1, B-capsids were produced, but the viral titer was reduced by 100- to 1000-fold, compared to the wild-type HSV-1 [47]. Moreover, the DNA packaging was impaired, which suggests that the scaffold is necessary to produce mature C-capsids.

#### 2.3.3. SCP

In HSV-1, the SCP is not required for B-capsid formation in the recombinant baculovirus/insect cell system, be it inside the intact cells or in their lysates (Table 2) [19,20,23]. Furthermore, the lack of SCP only modestly reduced viral replication in infected Vero cells or in an ocular mouse model, and C-capsids still formed [43]. The reduced viral replication in Vero cells was due to the reduced capsid recruitment of the auxiliary capsid protein UL25 (discussed in detail in the next section), which resulted in a capsid packaging defect; yet, C-capsids still formed [62]. In contrast, SCP was required for replication in the trigeminal ganglia [43], highlighting cell-specific requirements for SCP. While the role of the SCP during HSV-1 infection of the trigeminal ganglia is unclear, SCP did not appear necessary for capsid translocation from the mouse eye to the trigeminal ganglia. HSV-1 capsids lacking the SCP can acquire auxiliary capsid proteins [63], albeit in reduced amounts [62], and recent cryo-EM reconstruction of the HSV-1 capsid suggests that these proteins are UL17, UL25, and UL36 [12]. Therefore, capsids lacking SCP can still traffic within the infected cell because UL36, which is essential for capsid trafficking [64], can bind capsids even in the absence of SCP. It has been suggested that the SCP may, instead, have a role in reactivation from latency, which could explain the severe replication defect in trigeminal ganglia but not in Vero cells or the ocular model [43].

In VZV, the SCP was required for infection in a human skin xenograft model, and, in the absence of SCP in melanoma cell lines, only empty spherical capsids formed [56]. Therefore, even within the same subfamily, the relative contributions of the capsid proteins to capsid assembly and replication appear to differ.

### 2.4. Sub-Family-Specific Roles of Capsid Proteins in Gammaherpesvirus Assembly

#### 2.4.1. MCP and Triplexes

Capsid assembly in gammaherpesviruses has also been studied using the recombinant baculovirus/insect cell system. For either EBV or KSHV, overexpression of the MCP, triplexes, SCP, protease, and scaffold resulted in the assembly of B-capsids [21,22], which required the presence of the MCP and triplexes, similarly to HSV-1 [19,20,21,22]. These findings demonstrate similarities in the capsid assembly between the alpha- and gammaherpesvirus subfamilies. Interestingly, in KSHV, auxiliary capsid proteins can bind the B-capsids formed from the recombinant baculovirus/insect cell system [65]. This approach could provide a convenient way for studying how these proteins interact with the capsid and comparing these interactions across subfamilies, but such experiments have not yet been done.

#### 2.4.2. Protease and Scaffold

In EBV and KSHV, as in HSV-1, in the absence of both the scaffold and protease, only open crescent capsid structures could form in the recombinant baculovirus/insect cell system [19,20,21,22]. Likewise, in the absence of only the protease, capsids could still assemble in all three viruses [19,20,21,22]. The EBV capsids that formed in the absence of the protease were likely procapsids because they did not withstand centrifugal forces used during their isolation by sedimentation [21], similarly to HSV-1 procapsids [24]. Furthermore, only procapsids were formed in cells infected with either a protease-null KSHV or a KSHV containing a point mutation that eliminates protease activity [51], similarly to what was observed in HSV-1 protease-mutant viruses [30]. Collectively, these data point towards the importance of the protease in the procapsid angularization in both the alpha- and gammaherpesvirus subfamilies.

One notable difference in the assembly of both the EBV or KSHV capsids relative to HSV-1 is that in the recombinant baculovirus/insect cell system, only open-crescent capsid structures and no closed capsids, spherical or angularized, were observed for EBV and KSHV in the absence of a scaffold (Table 2) [21,22]. This implies that, despite containing the entire amino acid sequence of the scaffold, the protease is unable to act as the scaffold during gammaherpesvirus capsid assembly, at least not in the context of the recombinant baculovirus/insect cell system.

#### 2.4.3. SCP

Another notable difference from HSV-1 is that neither EBV nor KSHV capsids formed in the recombinant baculovirus/insect cell system in the absence of the SCP (Table 2) [21,22]. Likewise, capsid assembly was largely blocked in cells infected with SCP-null mutant KSHV [46], although some capsids, predominantly empty, could still form [45]. In contrast, HSV-1 did not require SCP for capsid assembly under any tested conditions, namely, in vitro, in insect cells expressing capsid proteins, or in either HSV-1 infected Vero cells or an ocular mouse model [19,20,23,43,62].

### 2.5. Sub-Family-Specific Roles of Capsid Proteins in Betaherpesvirus Assembly

#### 2.5.1. MCP and Triplexes

Both the MCP and the triplexes are required for HCMV replication in human primary foreskin fibroblasts [42], presumably due to their requirement for capsid assembly (Table 2). Although assembly experiments have not yet been performed for betaherpesvirus capsids, capsid assembly in all three subfamilies likely requires the MCP and triplexes, which is consistent with their roles as core capsid shell components.

#### 2.5.2. Protease and Scaffold

In cells infected with protease-null HCMV, angularized capsids with dense cores, which presumably correspond to uncleaved scaffold, formed and DNA packaging was severely reduced [50]. In contrast, in cells infected with protease-null HSV-1 or KSHV, DNA packaging was defective but, instead of angularized capsids, only spherical procapsids formed [30,49,51]. These findings suggest that the protease is required in all three subfamilies for DNA packaging but only in alpha- and gammaherpesviruses for the angularization of procapsids.

In cells infected with an HCMV mutant virus containing a point mutation in the scaffold that prevents interactions with the MCP (Table 2), mostly open-crescent-shaped structures were observed, along with some closed capsids of undetermined type [48]. This suggests that like HSV-1 protease [47], the HCMV protease can substitute for the scaffold even if poorly. In contrast, gammaherpesviruses appear to have a strict requirement for the scaffold, at least in the recombinant baculovirus/insect cell system.

#### 2.5.3. SCP

HCMV capsids can form in infected cells in the absence of the SCP but cannot recruit an essential tegument protein, pp150, resulting in the production of B-capsids [44]. Furthermore, the HCMV SCP is required for replication in human primary foreskin fibroblasts [42] and in human retina pigment epithelial cells [66], presumably because it is necessary for the formation of mature C-capsids. Therefore, the requirement for the SCP for capsid assembly and replication varies significantly across subfamilies.

### 2.6. Summary of Capsid Assembly across Subfamilies

Collectively, capsid assembly studies performed in vitro from purified capsid proteins or in insect cells overexpressing capsid proteins largely recapitulate findings from infected cells. In all subfamilies, capsids fail to form in the absence of the MCP or triplexes, which is consistent with their roles as core capsid shell components. Capsids also fail to form in the absence of both the scaffold and protease in alpha-, gamma-, and, likely, betaherpesviruses, although the experimental evidence for betaherpesviruses is lacking. In all subfamilies, the protease is required for the formation of C-capsids, likely because the DNA cannot be packaged until the scaffold is removed. The protease activity is important for angularization in cells infected with either HSV-1 [30,49] or KSHV [51], which suggests that the scaffold interactions with the capsid shell may somehow preclude angularization. The defect in angularization in the absence of the protease could be kinetic, however, because in the case of HSV-1, some B-capsids can form in vitro even in the absence of the protease, but this process is slow and inefficient [24,30]. In contrast, the protease activity is not required for capsid angularization in HCMV-infected cells [50], suggesting that in HCMV angularization can occur even in the absence of scaffold cleavage.

The role of SCP in capsid assembly appears to be the least conserved. In HSV-1, it is dispensable for capsid assembly in vitro or in the recombinant baculovirus/insect cell system [19,20,23] and for replication in certain cells types [43,62]. In HCMV, SCP is required for the formation of C-capsids but not the B-capsids [44]. Finally, in EBV and KSHV, the SCP is required for capsid assembly in the recombinant baculovirus/insect cell system [21,22] and in KSHV-infected cells [45,46]. These phenotypic differences between the subfamilies are, perhaps, not surprising considering that the sequence of SCP is the least conserved among the capsid proteins in human herpesviruses [8]. Further experiments are needed to identify the mechanisms underlying the observed phenotypic differences in capsid assembly.

## 3. Capsids Require Additional Capsid-Associated Proteins for Successful Viral Replication

Compared to most mammalian viruses, herpesviruses have very large genomes that range from ~125 to ~250 kbp [67]. The capsid shell, however, is relatively compact, with an average diameter of ~125 nm [10,12,29]. As the result, the internal pressure within the capsid reaches unusually high levels among biological systems. For example, the internal pressure within an HSV-1 capsid is ~20 atm [68], which is comparable to the pressure at ~620 feet underwater. To withstand such high pressure from the encapsidated genome, herpesviruses reinforce their capsids using auxiliary capsid proteins that also participate in other steps of viral replication, particularly those involving capsids.

The auxiliary capsid protein complexes have been collectively referred to by many names, including the C-capsid-specific component (CCSC) [69], the capsid vertex-specific component (CVSC) [70], and most recently, the capsid-associated tegument complex (CATC) [12]. For consistency across the subfamilies, we will refer to these complexes as the CATC. Recent advancements in cryo-EM imaging have yielded the C-capsid structures of nearly all human herpesviruses, including HSV-1 [12], HSV-2 [14], VZV [8], HCMV [13,16], HHV-6B [11], EBV [9,15], and KSHV [10,71]. Although not the focus of this review, the structures of the C-capsids from two animal herpesviruses, the pseudorabies virus [72] and the murine cytomegalovirus [73], have also been determined. These studies revealed both the similarities and the differences among the CATCs from different subfamilies and, in some cases, differences among the CATCs within the same subfamily. These differences likely reflect evolutionary adaptations due to differences in cellular tropism and genome size across subfamilies.

### 3.1. Alphaherpesviruses

#### 3.1.1. CATC Components, Capsid Location, and Occupancy

**Figure 3:**
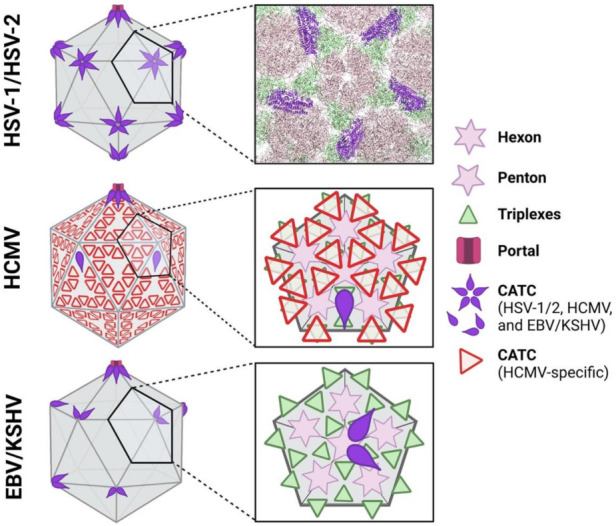
Comparison of the CATC location and occupancy on the capsid among the representatives of the three human herpesvirus subfamilies. In both alpha (HSV-1/2) and gammaherpesviruses (EBV/KSHV), the CATC (purple raindrops) is composed of three proteins (HSV1/2: UL17, UL25, and UL36; EBV: CVC1, CVC2, and LTP; KSHV: ORF32, ORF19, and ORF64) and is located at capsid pentonal vertices. CATC on HSV1/2 capsids is present at a full occupancy whereas CATC on gammaherpesvirus capsids is present at a lower occupancy. Unusually, HCMV has two types of CATCs. The first CATC type (purple raindrops) is the conserved complex of UL93, UL77, and UL48 (homologs of UL17, UL25, and UL36) present at the lowest occupancy and positioned slightly below pentons. The other CATC type (red triangles) is betaherpesvirus-specific and covers the entire capsid like a net. In all three subfamilies, the portals are fully occupied by the CATC. Insets show CATC location relative to the MCP hexons, MCP pentons, and triplexes. For clarity, the SCP is not shown. The HSV-1/2 inset shows the 3D structure of this region of the HSV-1 capsid (UL25 globular domains not shown; PDB: 6CGR), generated using Chimera [79]. This figure was created with BioRender.com, accessed on 17 September 2021.

**Figure 4:**
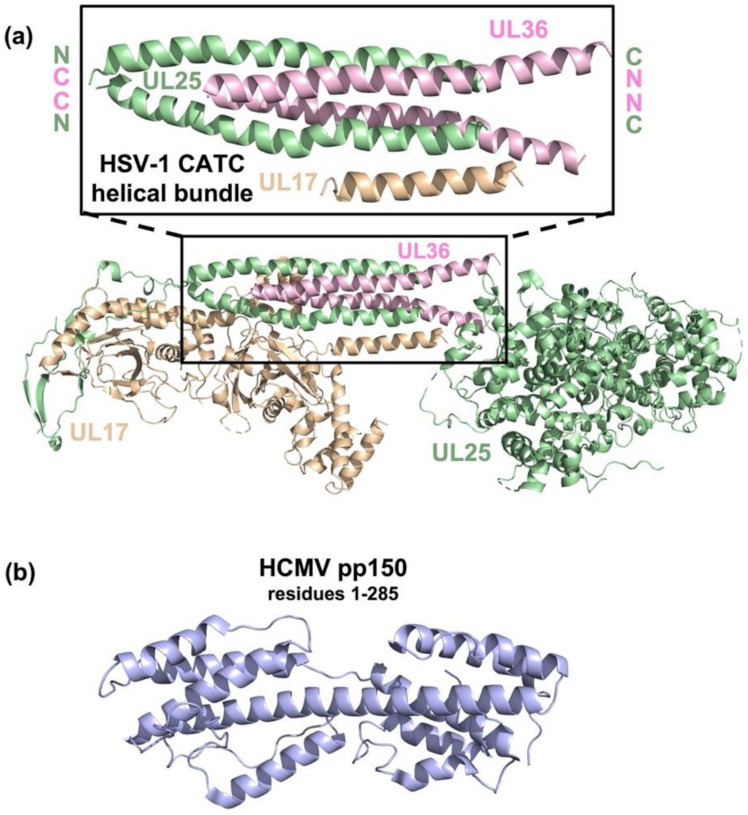
Comparison of the conserved CATC of HSV-1 and the betaherpesvirus-specific CATC of HCMV. (**a**) The structure of the HSV-1 CATC (UL17, UL25, and UL36) (PDB: 6CGR). The inset shows a closeup of the HSV-1 CATC helical bundle along with corresponding locations of N and C termini in each protein. (**b**) The structure of the HCMV CATC protein pp150 (PDB: 5VKU); Structures were visualized using PyMOL [88].

In the in situ structures of HSV-1 and HSV-2 C-capsids, the CATC is a heteropentamer composed of one copy of UL17, two copies of UL25, and two copies of UL36 (although only the C-terminal helix was resolved) [12,14]. Five copies of the CATC are arranged at each of the 11 pentonal capsid vertices (Figure 3), with the globular domains interacting with the penton and the triplexes to form a star-shaped crown. Helices from UL25 and UL36 form a four-helix bundle (Figure 4a) that projects outward from the globular domains (Figure 3). Recent in situ structures of the C-capsids from HSV-1 [74,75] and HSV-2 [76] also show the CATC at the portal vertex. However, the CATC is oriented somewhat differently at the portal compared to the other vertices.

In the in situ structure of the VZV C-capsid, only the UL17 (ORF43) and the UL25 (ORF34) homologs were resolved within the CATC [8] (see Table 1 for detailed nomenclature). The lack of the density for the UL36 homolog (ORF22) could be due to the relatively low resolution of the cryo-EM reconstruction, presumably caused by the reduced sample availability for data collection due to the tendency of VZV virions to associate with host cells. Considering that ORF22 associates with capsids in the nucleus, it is most likely present in the samples despite being absent from the cryo-EM reconstruction [77]. Compared to the HSV-1 and HSV-2 counterparts, the occupancy of the VZV CATC at the vertices was only ~50% [8]. To test the relative affinity of VZV CATC binding to capsids, virions were treated with detergent followed by increasing amounts of salt, and the ORF43 (UL17 homolog) protein was easily removed from the capsids after salt treatment. In contrast, HSV-1 UL17 remains bound to the capsid under the same conditions. Overall, these findings suggest that CATC from HSV-1, and most likely HSV-2, binds capsids with a higher affinity than its VZV counterpart. Surprisingly, purified VZV C-capsids did not contain any CATC, which could be due to the high centrifugation speeds used during purification that may have caused CATC to detach from the capsids. Therefore, one would expect C-capsids purified using a gentler centrifugation strategy [78] to contain CATC in amounts consistent with what is observed in the in situ structure. 

#### 3.1.2. Functional Roles of Alphaherpesvirus CATC Proteins

The roles of the alphaherpesvirus CATC proteins have been most extensively studied in HSV-1 [6]. Although UL17, UL25, and UL36 form a complex on the capsid, each serves a different role in viral replication. Both UL17 and UL25 are essential for DNA packaging [80,81,82,83,84,85], but only UL17 is required for genome cleavage [82]. While capsids still form in the absence of either protein, most of them lack genomes (B-capsids for UL17-null and A-capsids for UL25-null). Capsids isolated from cells infected with either a UL17-null or a UL25-null mutant of HSV-1 were found to be less stable compared to wild-type capsids, as determined by atomic force microscopy, suggesting both proteins also structurally reinforce the capsid [86,87].

UL36 is a large conserved tegument protein that is composed of folded domains interspersed with unstructured regions and serves as a major hub that connects the capsid to the tegument layer [89]. It has been studied most extensively in HSV-1 and PRV where it is essential for viral replication [90] and is involved in capsid trafficking to the nucleus [64,91,92], capsid docking at the nuclear pore [93], and the release of the viral DNA into the nucleus [94] (Figure 1). For example, both UL36 and its CATC partner UL25 remain bound to the capsid upon entering a new host cell [64] and can bind to nucleoporins [93,95]. Removal of the last three C-terminal residues of UL25 prevents genome release from the capsid [96]. The viral DNA is thought to be released through the capsid portal complex rather than through an additional exit route created at a different capsid vertex. The presence of the CATC at the portal vertex in the cryo-EM capsid reconstructions of HSV-1 and HSV-2 [74,75,76] supports its involvement in capsid docking at the nuclear pore and the genome release into the nucleus (Figure 1). The HSV-1 CATC has also been implicated in the selection of mature C-capsids during nuclear egress [84,97,98,99], but whether this property is conserved across all herpesvirus subfamilies is yet unclear.

All three VZV CATC components are required for viral replication [100] but may be less important for the structural integrity of the capsid than their HSV counterparts due to lower occupancy and weaker affinity for the capsid [8]. By analogy with the HSV CATC, the VZV CATC at the portal vertex would be expected to participate in genome delivery to the nucleus and DNA encapsidation, but these possibilities await experimentatal validation.

### 3.2. Gammaherpesviruses

#### 3.2.1. CATC Components, Capsid Location, and Occupancy

Gammaherpesviruses encode the CATC homologs of UL17, UL25, and UL36 (EBV: BGLF1 (CCV1), BVRF1 (CCV2), and BPLF1 (LTF); KSHV: ORF32, ORF19, and ORF64), and the C-capsid structures of both EBV and KSHV have CATC of the same composition as alphaherpesviruses, judging by the in situ cryo-EM structures [9,10,15] (Table 1). The CATCs on gammaherpesviral capsids also form the canonical helical bundles that radiate outward from the globular domains at the vertices even though their orientations differ slightly. A top-down view shows that the globular domains of the CATC are located on the right side of the helical bundles in KSHV but on the left side in EBV [9]. The CATC occupancy is significantly lower in EBV (~20%) and KSHV (~40%) compared to the full occupancy in HSV-1 and HSV-2 (Figure 3) [12,14]. Moreover, KSHV follows the “portal-side equatorial rule” such that the CATC preferentially binds the vertices near the portal [10], whereas EBV has a generally random arrangement [9]. The CATC stoichiometry also differs in that, on average, only one or two copies of the CATC are found at EBV and KSHV capsid vertices [9,10], respectively, in contrast to the full set of five copies found at alphaherpesvirus capsid vertices (Figure 3) [8,12,14]. Nonetheless, in both EBV and KSHV, portals have full CATC occupancy.

The lower CATC occupancy in gammaherpesviruses – particularly in KSHV, where the extent of CATC occupancy varied between virions collected for data analysis [10] – could, potentially, be due to differences in cellular tropism. HSV-1/2 are neurotropic viruses, and their capsids must traffic long-range along axons, which requires the CATC component UL36 [64]. A full UL36 occupancy on the capsid could be beneficial to sustained transport of HSV-1/2 capsids, for example, by providing multiple binding sites for the host motor proteins. By contrast, EBV and KSHV are lymphotropic viruses, so while their capsids also engage host motor proteins, they do not need to traffic long-range, which could eliminate the need for a full CATC set. Further, KSHV ORF64, like its HSV-1 UL36 homolog, serves as the central tegument hub and coordinates secondary envelopment [101], but this process may require fewer capsid-bound copies of ORF64, perhaps, due to differences in tegument composition. These considerations could explain how KSHV capsids remain functional despite the reduced CATC occupancy.

#### 3.2.2. Functional Roles of Gammaherpesvirus CATC Proteins

Little information is available regarding the functions of the gammaherpesvirus CATC proteins in viral replication. Given the overall structural similarity between the alphaherpesvirus and gammaherpesvirus CATC structures, some functions may overlap. One conserved function most likely involves capsid docking at the nucleus and genome delivery. KSHV capsids lacking a portal complex do not efficiently dock at the nucleus [102], likely because the portal vertex also lacks the CATC. CATC proteins may also have subfamily-specific functions. Further mutational analyses are needed to elucidate the roles of gammaherpesvirus CATC proteins in viral replication.

### 3.3. Betaherpesviruses

#### 3.3.1. CATC Components, Capsid Location, and Occupancy

Betaherpesviruses encode the CATC homologs of UL17, UL25, and UL36 (UL93, UL77, and UL48, respectively), as well as another CATC protein, HCMV pp150 (HHV-6B U11) (Table 1) [11,13], that has no homologs in either alpha- or gammaherpesviruses and appears specific to betaherpesviruses [44]. HCMV pp150 is a ~1000-amino-acid protein predicted to have one helical domain followed by a largely unstructured region. Indeed, only the mostly helical ~1/3 of pp150 was resolved in the cryo-EM reconstructions (Figure 4b) [13]. HHV-6B U11 is predicted to be more structured; yet, similarly to HCMV pp150, only the N-terminal 1/3rd was resolved in the cryo-EM reconstruction [11]. Three copies of HCMV pp150 form a triangular structure [13], whereas two copies of HHV-6B pU11 form a V-shape [11]. Both structures bridge the MCPs and the triplexes (Figure 3).

A recent cryo-EM reconstruction of the HCMV capsid in situ revealed the presence of the conserved CATC, composed of UL93, UL77, and UL48, at the pentonal capsid vertices and the portal (Figure 3) [16]. These CATC components form the canonical helical bundle also observed in the alpha- and gammaherpesvirus subfamilies, except that only one copy of the globular head domain of UL77 (HSV-1 UL25 homolog) was resolved. This conserved CATC was present at an even lower occupancy on HCMV capsids than on EBV or KSHV capsids [12,14], with only ~12 copies/capsid [16]. This reduced occupancy is offset by the large copy number of the betaherpesvirus-specific CATC protein pp150 (~900) on the HCMV capsid. While only U11 has been resolved on the HHV-6B capsid to date [11], the HHV-6B UL93/UL77/UL48 homologs are likely also present on the capsid.

Unlike the alpha- and gammaherpesvirus CATCs, the betaherpesvirus CATCs (both pp150 and the UL93/UL77/UL48 complex) form a net around the capsid (Figure 3). Such an arrangement could serve to increase capsid stability allowing it to contain a larger genome that, presumably, generates higher internal pressure. Indeed, HCMV, which has the largest genome among all human herpesviruses (~235 kbp) [103], has the most CATC copies. In contrast, HHV-6B, which has a smaller genome (~165–170 kbp), also has fewer CATC copies [104]. Capsids of alpha- and gammaherpesviruses, which contain genomes of similar size to HHV-6B (~150–170 kbp) [105,106], have even fewer CATC copies, but the different composition and placement of the CATCs precludes direct comparisons.

Recent theoretical models estimated the internal capsid pressures of human herpesviruses and proposed that HCMV capsids are under much less pressure (~45 atm) compared to EBV capsids (~90 atm) despite having larger genomes [86]. It has also been shown that the HCMV genome is packaged more densely compared to HSV-1 [107]. These observations suggest that other factors, such as variations in electrostatic forces of the packaged genome, may influence internal capsid pressure. These factors may vary across herpesvirus subfamilies. Experiments measuring herpesvirus capsid stability in the presence and absence of CATC components from each subfamily would help clarify this issue.

#### 3.3.2. Functional Roles of Betaherpesvirus-Specific CATC Proteins

Most functional information regarding the role of betaherpesvirus CATCs comes from studies on HCMV pp150. This tegument protein has multiple roles in viral replication, including capsid stability and trafficking [108,109], DNA packaging [44], and secondary envelopment [110], and is also a cell-cycle-dependent restriction factor [111]. Many of these roles resemble the roles of the HSV-1 CATC components, some of which are essential for DNA packaging (UL17 and UL25) or capsid trafficking (UL36). In principle, pp150 could subsume some of the functions of these proteins. For example, the unstructured C-terminus of pp150 could interact with a variety of host and viral proteins during infection, and its additional functions await to be discovered.

#### 3.3.3. Functional Roles of the Betaherpesvirus CATC Proteins Conserved in Alpha- and Gammaherpesviruses

HCMV UL93 and UL77 (HSV-1 UL17 and UL25 homologs, respectively) were recently observed in the structure of the HCMV capsid [16], confirming previous immunoblot analyses of HCMV capsids from infected cell nuclei that identified UL93 and UL77 as capsid components [112] (Table 1). Additionally, in the absence of either protein, the DNA cleavage and the formation of C-capsids are blocked, and only B-capsids form [112,113], which implicates UL93 and UL77 in DNA cleavage and packaging. Indeed, these two proteins are bound at the HCMV capsid portal [16]. It is yet unknown if these findings also extend to HHV-6B.

HCMV UL48 (a homolog of HSV-1 UL36) is thought to have roles similar to its HSV-1 UL36 counterpart (described above), which include capsid trafficking through the cell, capsid tegumentation, and secondary envelopment [114,115], and also localizes to both the nucleus and the cytoplasm [116]. Viral replication in human foreskin fibroblasts was reduced 100-fold compared to wild-type when UL48 nuclear localization was blocked, which suggests that UL48 is involved in viral replication steps in the nucleus, by an unknown mechanism. Similarly to HSV-1 UL36, UL48 could associate with capsids prior to nuclear egress. Cryo-electron tomography and biochemical analyses of HCMV virions revealed that UL48 and pp150 form the innermost layer of the tegument [117], and both were resolved in the recent HCMV cryo-EM capsid reconstruction [16]. Although these proteins do not appear to interact on the capsid, both are large proteins with flexible regions, unresolved in the cryo-EM reconstructions, that could, in principle, interact with one another in the virion tegument layer. Future advancements in cryo-EM technology may allow for the visualization of these potential interactions.

## 4. Conclusions and Future Directions

Human herpesviruses have evolved unique strategies for infecting and persisting in a variety of cell types, yet many of these strategies are still not fully understood. A deep understanding of herpesvirus assembly is essential for developing novel therapeutic and preventative measures against these common pathogens. The bulk of the existing knowledge of the capsid assembly is based on the studies in HSV-1 in several experimental systems, including in vitro assembly. Further studies of the HSV-1 capsid assembly, along with their extension to the beta- and gammaherpesviruses, are needed to answer lingering questions such as the following:

- Can alternative, gentler centrifugation strategies be developed to isolate larger quantities of intact virions and capsids for in-depth studies?
- What triggers besides protease-mediated scaffold cleavage are required for DNA packaging in infected cells?
- How much does the size of the genome influence the internal capsid pressure, and does this influence CATC occupancy?

Identifying the similarities and the differences among herpesviruses may also reveal potential targets for pan-herpesvirus therapeutics. To our knowledge, there is only one known capsid assembly inhibitor, which prevented VZV capsid formation by altering the localization of the MCP in VZV-infected cells [118]. It is yet unclear whether this inhibitor is effective in vivo or against other human herpesviruses. Additional capsid assembly inhibitors, targeting the portal complex, have been identified in HSV-1 and VZV, but a majority of these findings await in vivo testing (reviewed in [119]). Therefore, further investigation of the mechanisms of capsid assembly among subfamilies is needed to fully test the therapeutic potential of this essential stage in herpesvirus replication.

## Figures and Tables

**Table 1:** Gene and protein names for each capsid component from human herpesviruses with known capsid structures.

	Alpha						Beta				Gamma			
	HSV-1		HSV-2		VZV		HCMV		HHV-6		EBV		KSHV	
	Gene	Protein	Gene	Protein	Gene	Protein	Gene	Protein	Gene	Protein	Gene	Protein	Gene	Protein
**Portal**	UL6	UL6	UL6	UL6	ORF54	ORF54	UL104	Portal	U76	Portal	BBRF1	BBRF1	ORF43	ORF43
**MCP**	UL19	VP5	UL19	VP5	ORF40	ORF40	UL86	MCP	U57	MCP	BcLF1	BcLF1	ORF25	ORF25
**SCP**	UL35	VP26	UL35	VP26	ORF23	ORF23	UL48a (UL48.5)	SCP	U53	SCP	BVRF3	BFRF3	ORF65	ORF65
**Triplex 1**	UL38	VP19c	UL38	VP19c	ORF20	ORF20	UL46	mCP-BP	U29	Triplex 1	BORF1	BORF1	ORF62	ORF62
**Triplex 2**	UL18	VP23	UL18	VP23	ORF41	ORF41	UL85	mCP	U56	Triplex 2	BDLF1	BDLF1	ORF26	ORF26
**CATC**	UL25	UL25	UL25	UL25	ORF34	ORF34	UL77	UL77	U50	U50 *	BVRF1	CVC2	ORF19	ORF19
	UL17	UL17	UL17	UL17	ORF43	ORF43	UL93	UL93	U64	U64 *	BGLF1	CVC1	ORF32	ORF32
	UL36	UL36	UL36	UL36	ORF22	ORF22	UL48	UL48	U31	U31 *	BPLF1	LTP	ORF64	ORF64
**CATC (beta only)**	--	--	--	--	--	--	UL32	pp150	U11	U11	--	--	--	--

* Proteins were not identified as CATC components on HHV-6 capsids.

## Data Availability

Not applicable.
